# Supplementary figures and images for: Identification of MDK as a Hypoxia- and Epithelial–Mesenchymal Transition-Related Gene Biomarker of Glioblastoma Based on a Novel Risk Model and In Vitro Experiments
Source: Biomedicines. 2024 Jan 1;12(1):92. doi: 10.3390/biomedicines12010092 (PMC10813330; doi:10.3390/biomedicines12010092)

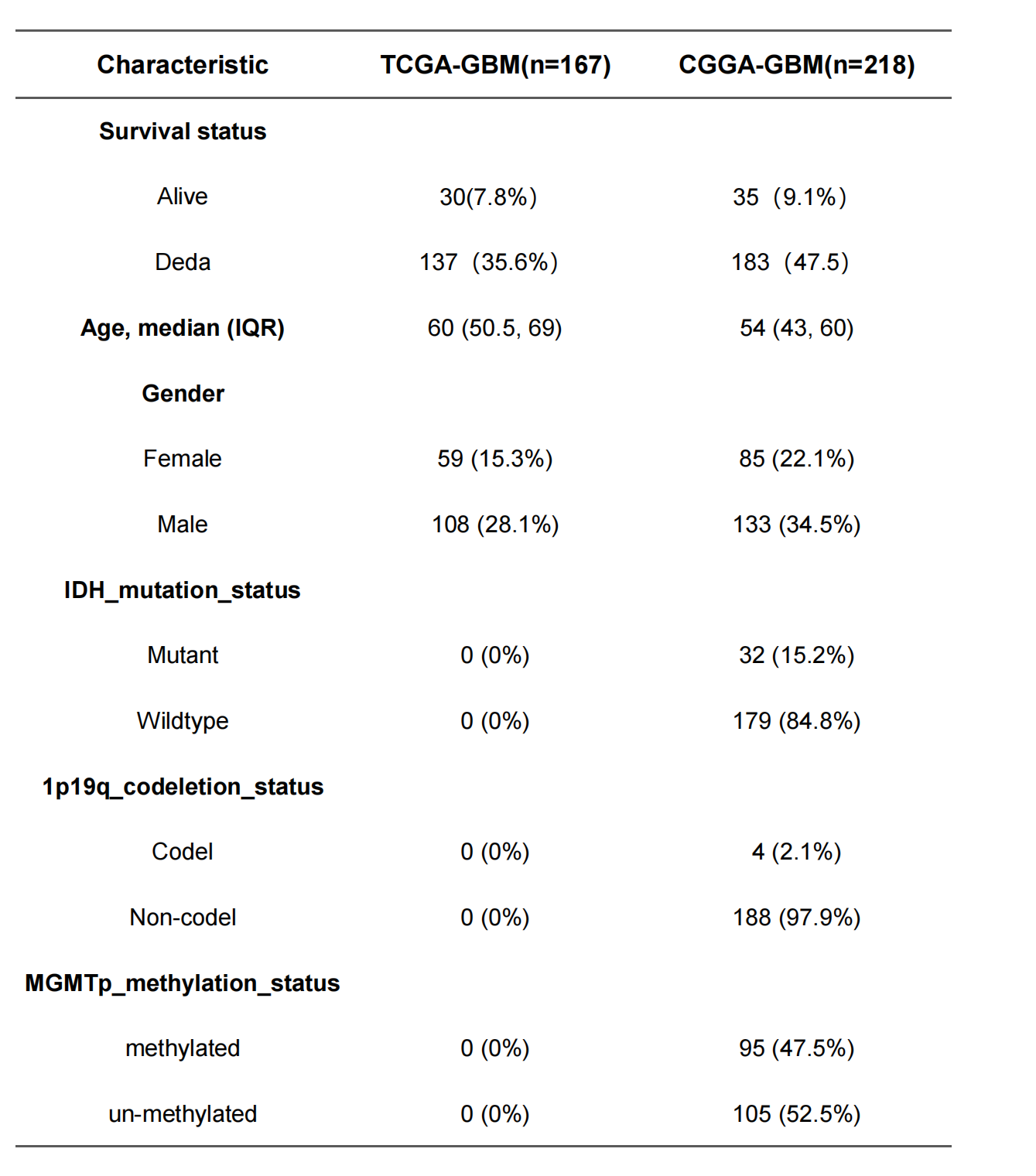

Supplement: Supplementary file 1 [file biomedicines-12-00092-s001.zip › biomedicines-2646744-supplementary/Supplementary Table S1.tif]
